# Fluoroquinolone Resistance among Clonal Complex 1 Group B* Streptococcus* Strains

**DOI:** 10.1155/2016/6403928

**Published:** 2016-07-31

**Authors:** Alefiya Neemuchwala, Sarah Teatero, Samir N. Patel, Nahuel Fittipaldi

**Affiliations:** ^1^Public Health Ontario Laboratory, Toronto, ON, Canada M5G 1M1; ^2^Department of Laboratory Medicine and Pathobiology, Faculty of Medicine, University of Toronto, Toronto, ON, Canada M5S 1A1

## Abstract

Fluoroquinolone resistance in group B* Streptococcus* is increasingly being reported worldwide. Here, we correlated fluoroquinolone resistance with mutations in* gyrA*,* gyrB*,* parC*, and* parE* genes, identified by mining whole-genome sequencing (WGS) data of 190 clonal complex 1 group B* Streptococcus* strains recovered from patients with invasive diseases in North America. We report a high prevalence of fluoroquinolone resistance (12%) among GBS strains in our collection. Our approach is the first step towards accurate prediction of fluoroquinolone resistance from WGS data in this opportunistic pathogen.

## 1. Introduction

Group B* Streptococcus* (GBS) causes neonatal sepsis and meningitis and is increasingly recognized as the causative agent of opportunistic infections in adults, particularly the elderly and those immunocompromised [1]. Ten GBS serotypes (Ia, Ib, and II to IX) have been described [2]. Historically, serotype V and, more recently, serotype IV have been associated with adult invasive GBS disease in North America [3–5]. Most serotype V strains isolated in this continent from the blood of adult patients with invasive GBS disease belonged to the multilocus sequence typing (MLST) sequence type (ST) 1 [3], whereas North American serotype IV isolates from adult invasive infections predominantly belonged to ST459 [4, 5]. These two STs are grouped in MLST clonal complex (CC) 1. Although penicillin continues to be the first antimicrobial choice for treatment, recent reports have described decreased susceptibility in some GBS isolates [6, 7]. Resistance to other agents such as erythromycin, clindamycin, and tetracycline is also common among invasive GBS strains [3–5]. Fluoroquinolones are another important antibiotic class commonly used in adults to treat infections [8, 9]. Resistance to fluoroquinolones among GBS strains has also been observed globally [10–12].

Phenotypic assays are the method of choice for determining drug susceptibility. However, nucleic acid-based tests detecting the presence of resistance genes and/or mutations in key genes leading to resistance have long proven useful for resistance prediction and elucidation of resistance mechanisms [8, 10, 13]. In recent years, nucleic acid-based detection systems have been impacted by the advent of relatively inexpensive whole-genome sequencing (WGS). Several bioinformatics tools permitting prediction of antimicrobial resistance encoded by acquired genes (i.e., those genes which can be either present in or absent from the genome of any particular strain) have been developed [13, 14]. Molecular prediction of fluoroquinolone resistance requires amplification and sequencing of genes* parC*,* parE*,* gyrA*, and* gyrB* [15]. Acquisition of mutation information by direct inspection of WGS short-read data obtained using the most common Illumina sequencing technology is challenging, as these genes are present in all strains, and no consistent database of known mutations is available.

## 2. Materials and Methods

### 2.1. Bacterial Strains

We tested 190 clonal complex 1 (85 serotype V ST1 and 105 serotype IV ST459) isolates that were recovered from patients with invasive infections in Canada as part of laboratory surveillance from 2009 to 2013 (Table 1). These isolates have been described earlier [3–5]. Isolates were grown on Columbia Blood agar at 37°C in the presence of 5% CO_2_.

### 2.2. Antibiotic Susceptibility Testing

Minimum inhibitory concentrations (MICs) for levofloxacin, ciprofloxacin, and norfloxacin were determined using the agar dilution method. The levofloxacin MIC results were interpreted as per Clinical and Laboratory Standards Institute (CLSI) guidelines [16], whereas norfloxacin and ciprofloxacin MIC results were interpreted based on previous studies [8, 9, 17].

### 2.3. Whole-Genome Sequencing and Phylogenetic Analysis

We used paired-end (150 nt + 150 nt or 101 nt + 101 nt) reads obtained using Illumina MiSeq or HiSeq instruments. NCBI Sequence-Read Archive accession numbers for WGS have been reported previously [3–5]. To evaluate the feasibility of predicting resistance to fluoroquinolones in GBS directly from short-read genome data, we first searched the literature for articles reporting mutations in genes* parC*,* parE*,* gyrA*, and* gyrB *that have been associated with fluoroquinolone resistance and created a working database of known mutations conferring resistance to this antimicrobial class. Next, we interrogated the 190 GBS genomes in our collection for mutations in* parC*,* parE*,* gyrA*, and* gyrB*. Briefly, we aligned the Illumina short-reads against reference strains (strain NGBS061 for ST459 isolates and strain SGBS001 for ST1 isolates, GenBank accession numbers CP007631.2 and CP010867.2, resp.) and identified polymorphisms using the variant ascertainment algorithm (VAAL) [18]. We also investigated the presence of genes encoding putative efflux pumps which might mediate fluoroquinolone resistance. To this end, we first generated* de novo* genome assemblies for all 190 strains using the A5 pipeline [19], followed by BLAST comparisons using the nucleotide sequences of known efflux pumps as queries.

## 3. Results

Previous studies have shown that CC1 GBS is a group of organisms predominantly associated with invasive diseases in adults [3–5]. Most of the isolates used here were recovered from the blood of adults and older adult patients (Table 1). Previous studies which evaluated in part the 190 GBS isolates in our collection reported highly prevalent resistance to erythromycin, clindamycin, and tetracycline [3–5]. Macrolide resistance was mostly attributed to the presence of* ermB*,* ermTR*, and/or other genes, while tetracycline resistance was associated with* tetM* genes [3–5]. Analysis of short-read WGS data revealed that 22 CC1 isolates had nonsynonymous single-nucleotide polymorphisms (SNPs) in genes* parC*,* parE*,* gyrA*, and* gyrB* (Table 2). All strains with a wild-type (WT)* parC*,* parE*,* gyrA*, and* gyrB* allele (defined as the DNA sequence of the allele found in the reference strain genome) were susceptible to levofloxacin (MIC < 1 mg/L), norfloxacin (MIC < 4 mg/L), and ciprofloxacin (MIC < 0.125 mg/L). Mutations in* parC* predominated among GBS strains exhibiting fluoroquinolone resistance. The predicted amino acid (aa) substitutions at positions 79 and 83 of ParC correlated with increased MICs to ciprofloxacin and levofloxacin. Four of the ST1 isolates with aa substitutions in ParC also had aa substitutions in GyrA, at aa positions 85 (*n* = 3) and 81 (*n* = 1). All of these isolates exhibited very high MICs to fluoroquinolones (Table 2). Thus, our data supports the previous observation that single point mutations in specific aa residues of ParC are sufficient to cause high-level resistance against norfloxacin and ciprofloxacin and to reduce susceptibility against levofloxacin [8]. However, the presence of mutations in both* parC* and* gyrA* resulted in very high level of resistance (MICs > 32 mg/L) of the strains to all fluoroquinolones including levofloxacin. We found that a single point substitution in ParC (S79F) in 3 different isolates leads to high level resistance (MIC > 32 mg/L) to norfloxacin and intermediate resistance to ciprofloxacin (MIC 4 mg/L). Thus, we speculate that aa substitution at residue 79 impacts more severely the binding of fluoroquinolones than substitutions at aa position 83. None of the single point mutations at aa position 83 of ParC led to the development of high level resistance to levofloxacin and ciprofloxacin. In addition, none of the other single mutations in the* gyrA* gene that we identified led to fluoroquinolone resistance* per se*. Similarly, most mutations in* gyrB *did not lead to development of resistance. However, a single aa substitution (E224G) in GyrB resulted in an MIC of 16 mg/L for norfloxacin in a single ST1 isolate. We also identified several mutations in the* parE* gene. We discovered that a Q444K substitution in ParE led to ciprofloxacin resistance. Another substitution (R449S) in ParE also led to low level resistance to norfloxacin. Finally, we identified one GBS strain without mutations in any of* parC*,* parE*,* gyrA*, and* gyrB *genes which was highly resistant to norfloxacin (MIC > 32 mg/L) and ciprofloxacin (MIC = 8 mg/L) but susceptible to levofloxacin. BLAST analysis of the genome of this strain identified the presence of a gene that is very similar to* S. pneumoniae pmr*, a known norfloxacin efflux pump [20]. BLAST comparisons did not identify this efflux pump in any of the other 189 GBS strains.

## 4. Discussion

GBS causes neonatal diseases and is also responsible for an increasing number of invasive infections in adults. The serotype V ST1 and the serotype IV ST459 GBS isolates included in this study represent two emerging GBS groups associated with adult invasive GBS infections in North America. Previous work has shown that rates of resistance to tetracyclines, erythromycin, and clindamycin among GBS strains in general and in strains included in our collection in particular are relatively high [3–5]. Here, we report a relatively high rate (12.4%) of fluoroquinolone nonsusceptible isolates and high resistance to levofloxacin (2%) among serotypes V and IV CC1 GBS strains causing adult invasive diseases. A previous investigation described low (0.7%) fluoroquinolone resistance rates among *β*-hemolytic streptococci in North America [11], but that study did not discriminate among GBS subtypes. On the other hand, high rates of fluoroquinolone resistance have been observed in GBS isolates (mostly serotype III or Ib ST19 strains) from Japan (18.4%), Taiwan (4.8%), and China (37.5%) [12]. Although resistance to fluoroquinolones appears to be on the rise in Canada, the rates reported here are lower than those in these Asian countries, which may be reflective of differences in antimicrobial usage practice in these different geographies. Interestingly, recent reports have also described reduced susceptibility to penicillin mediated by mutations in penicillin-binding protein PBP2X among some fluoroquinolone-resistant GBS from Japan [6, 7]. However, we did not observe mutations in PBP2X among fluoroquinolone-resistant GBS isolates in our collection.

We found that high level of fluoroquinolone resistance correlated with mutations in both* parC* and* gyrA* genes. Varying levels of resistance were observed when single amino acid substitutions occurred in either* parC*,* gyrA*,* parE*, or* gyrB*. Resistance to fluoroquinolones can arise from mutations in the so-called quinolone resistance determining region (QRDR) of* parC*,* parE*,* gyrA*, and* gyrB* genes but can also result from protection of quinolone binding to* gyrA* and topoisomerase and/or the action of efflux pumps, which are sometimes encoded in extra chromosomal genetic elements [15]. Interestingly, although norfloxacin is no longer used in treatment, we also identified that a single strain with no mutations in these four genes but which was resistant to norfloxacin possessed a copy of a gene encoding a known efflux pump described in other species [20]. A recent study from South Korea also reported norfloxacin resistance in GBS isolates mediated by the action of efflux pumps [17].

Several tools permitting rapid detection of antimicrobial resistance encoded by acquired genes (i.e., those which can be either present in or absent from the genome of any particular strain) have been developed [13, 14]. Our findings suggest that, provided a consistent database of known mutations, fluoroquinolone resistance can be predicted from WGS data with relative ease. WGS of hundreds of bacterial organisms at diagnostic and public health laboratories is now a reality. Working towards the development of curated databases linking genotypic information, such as the mutations described here, with phenotypic results is crucial to achieve the goal of predicting antimicrobial resistance from WGS data rapidly and accurately.

One limitation of our study is that our collection of organisms is temporally restricted (isolates recovered 2009–2013), thus hampering investigations into the evolution of mutations associated with fluoroquinolone resistance. However, we identified that most mutations related to fluoroquinolone resistance appeared only once, consistent with highly clonal ST1 and ST459 GBS organisms that may be undergoing continuous clonal expansion [3–5]. We also did not observe any conclusive evidence of strain clustering based on mutations in genes associated with fluoroquinolone resistance nor any obvious temporal associations (data not shown). Another limitation is that we do not know whether the patients from whom isolates were recovered had been prescribed fluoroquinolones. However, even if fluoroquinolones might not be commonly used to treat GBS adult infections in Canada, it can be speculated that increased fluoroquinolone utilization in treatment of respiratory and urinary tract infections may be contributing to selection of resistant strains among the commensal GBS population in adults [9]. Interrogating WGS data to identify mutations leading to resistance in GBS is challenging as there is no database of known mutations. Susceptibility testing is the gold standard for identifying resistance among clinical strains. Our study provides further evidence on linking resistance to mutations in* parC*,* parE*,* gyrA*, and* gyrB* genes and demonstrates the feasibility of applying WGS data to identify antimicrobial resistance for such genes.

## Figures and Tables

**Table 1:** Clonal complex 1 group B *Streptococcus* strains used in this study.

Age group^a^	Sequence type	Number of strains	Source of isolation			Year of isolation						Macrolide resistance gene			Tetracycline resistance gene
			Blood	Joint fluid	Other^b^	2009	2010	2011	2012	2013	2014	*ermTR*	*ermB*	*mefA*	*tetM*
EOD (*n* = 6)	1	3	2	—	1	—	1	2	—	—	—	—	1	—	2
	459	3	1	—	2	—	—	—	1	2	—	3	—	—	3

LOD (*n* = 1)	459	1	1	—	—	—	—	—	1	—	—	—	—	—	1

Child (*n* = 3)	459	3	2	—	1	—	1	1	1	—	—	3	—	—	2

Adult (*n* = 74)	1	19	16	3	—	—	5	8	6	—	—	3	2	1	15
	459	55	23	3	29	—	13	12	15	11	4	51	—	—	48

Older adult (*n* = 106)	1	63	61	1	1	2	26	23	12	—	—	16	13	2	51
	459	43	30	4	9	1	4	16	11	8	3	41	—	—	37

^a^Isolates were collected from neonates with early onset disease (EOD) (less than 7 days of age); infants with late onset disease (LOD) (7 to 90 days of age); children (aged more than 90 days to 18 years); adults (19 years to 59 years); and older adults (more than 60 years of age).

^b^Samples collected from normally sterile sites other than blood or synovial fluid.

**Table 2:** Characteristics of mutations observed in GyrA, GyrB, ParC, and ParE among ST459 and ST1 group B *Streptococcus* isolates used in this study.

ST	Number of isolates	Predicted amino acid substitution				Antibiotic^a^			MIC (mg/L)			Year	Reference
		GyrB	GyrA	ParC	ParE	LVX	NOR	CIP	LVX	NOR	CIP		
1	3		E85K	S79F		R	R	R	>32	>32	>32	2010, 2012	[7, 10]
1	1		S81L	S79F		R	R	R	>32	>32	>32	2011	[7, 9]
1	1	V79I				S	R	NS	1.5	8	1	2010	This study
1	1	E224G				S	R	NS	1.5	16	1	2010	This study
1	1	E566D				S	R	NS	1	4	1	2012	This study
1	1		G717S			S	R	NS	1.5	4	1	2010	This study
1	1			S79F		I	R	R	4	>32	4	2010	[7, 10, 11]
1	1			D83G, D806G		I	R	R	4	16	2	2011	[7, 10]
1	1				R449S	S	R	NS	2	16	1	2010	This study
1	1				Q444K	S	R	R	1.5	8	8	2011	This study
1	1				A218T	S	R	NS	1.5	4	1	2012	This study
459	1	S412L				S	R	NS	1	8	1	2012	This study
459	2		R474H			S	R	NS	1	4	1	2010, 2012	This study
459	1			D83V		I	R	R	4	32	4	2012	This study
459	1			D83Y		I	R	R	4	>32	4	2012	[10]
459	2			S79F		I	R	R	4	>32	4	2011	[7]
459	1				V627I	S	R	R	1.5	8	2	2010	This study
459	1				V206I	S	R	NS	1	4	1	2012	This study

^a^R: resistant, S: susceptible, I: intermediate resistance, and NS: increased MIC. For levofloxacin (LVX), MIC of ≥8 mg/L: resistant, 4 mg/L: intermediate resistant, and ≤2 mg/L: susceptible. For norfloxacin (NOR), MIC of ≥4 mg/L: resistant; ≤1 mg/L: susceptible. For ciprofloxacin (CIP), MIC of >2 mg/L: resistant; ≤0.125: susceptible. Values which were in the range of 0.125 to 2 were considered as having reduced susceptibility (NS).
